# Socio-cultural practices and experience of mothers’ post stillbirth and newborn death: a population-based perspective from India

**DOI:** 10.1186/s12884-024-06906-0

**Published:** 2024-11-25

**Authors:** Moutushi Majumder, G Anil Kumar, Sarah Binte Ali, Sibin George, Siva Prasad Dora, Md. Akbar, Shuchi Sree Akhouri, Sweta Kumari, Tanmay Mahapatra, Rakhi Dandona, ENHANCE 2020 Team, Moutushi Majumder, G Anil Kumar, Sibin George, Siva Prasad Dora, Md. Akbar, Rakhi Dandona, Arpita Paul, Arup Kumar Das, Lalit Dandona, Vimal Kumar, Debrupa Bhattacharjee, Dinesh Bhatt

**Affiliations:** 1 Public Health Foundation of India, New Delhi, India; 2 Piramal Swasthya Management and Research Institute, Hyderabad, India; 3 Institute for Health Metrics and Evaluation, University of Washington, Seattle, USA

**Keywords:** Social support, Negative experience, Stillbirth, Neonatal death, Bihar, India, Post-partum maternal health, Cultural practices

## Abstract

**Introduction:**

We report on post stillbirth and newborn death socio-cultural experience of women from a population-based representative sample in the Indian state of Bihar.

**Methods:**

A state-representative sample of 7,270 births between July 2020 and June 2021 was sampled, including 582 stillbirths and 831 newborn deaths. Detailed confidential interviews were conducted with the consenting women with stillbirth and newborn death to understand their post-birth experience.

**Results:**

A total of 501 (86.1% participation) women with stillbirth and 717 (86.3% participation) with neonatal death provided interview. Able to talk to someone about their baby and receiving support to cope with their loss were reported by 369 (74.2%) and 398 (80.2%) women with stillbirth; these proportions were 76.7% and 77.3% for women with newborn deaths, respectively. More than 80% of these women reported spouses as their main source of support. At least one negative experience was reported by 150 (30.9%) and 233 (32.5%) women with stillbirth and newborn death, respectively. The most commonly reported negative experience was receiving insensitive/hurtful comments about the baby (18.6% for stillbirth and 20.4% for newborn deaths), followed by being blamed for the baby’s death (14.3% for stillbirths and 15.0% for newborn deaths). The majority of women reported being verbally abused by the mother-in-law for both stillbirth (24, 63.2%) and newborn death (49, 64.5%); while 48 (67.6%) and 66 (61.7%) women were blamed by the mother-in-law for stillbirth and neonatal death, respectively. Most women with stillbirth (72.7%) and with neonatal death (77.1%) were asked to forget about their babies as a means to cope with their loss. Naming, seeing, and holding the stillborn were reported by 56 (11.2%), 229 (45.9%), and 64 (12.8%) women with a stillborn.

**Conclusion:**

With one-third women with adverse birth outcome reporting negative experience, this translates into a significant number of women in India as it accounts for high numbers of stillbirths and newborn deaths globally. These population-based data can facilitate in designing interventions to improve post-partum experience for women with adverse birth outcomes in India.

**Supplementary Information:**

The online version contains supplementary material available at 10.1186/s12884-024-06906-0.

## Background

The loss of a child through stillbirth or neonatal death is a devastating experience that impacts women and families worldwide. Social support and kinship are considered essential factors in helping bereaved mothers cope with their loss and grief [1–5]. However, compounding the journey of grieving mothers is the unfortunate reality of negative social experiences emanating from both their families and the society at large [6–8]. Much information on such experiences is available on the experience of women with stillborn babies and not with a newborn death, and is available predominately around the behaviour of health care providers from developed country settings [8–14]. Stillbirth-related stigma has been documented as labelling, stereotyping, separation, status loss and discrimination, as well as power imbalance to influence in various settings [7, 15, 16].

India continues to account for significant numbers of stillbirths and neonatal deaths globally [17–19]. India, where socio-cultural practices play a significant role in shaping maternal experiences [20, 21], there is a paucity of research examining the socio-cultural dimensions that influence how mothers navigate grief post adverse birth outcome. Studies have documented how the health care providers manage grieving mothers, [22–24] and three qualitative studies with relatively small sample sizes have shed light on maternal grief and have identified stillbirth as a major cause of psycho-social morbidity, including high rates of depressive symptoms, anxiety, post-traumatic stress and suicidal ideation [8, 25, 26]. Population-based understanding of socio-cultural practices and experiences of mothers’ post-stillbirth and neonatal deaths in India is not readily available. In this background, we present findings on the socio-cultural dimensions and experience of women post-stillbirth and newborn death in a representative sample from the Indian state of Bihar, which has reported one of the highest neonatal mortality rates in the country [18] and a significant number of stillbirths [27].

## Methods

### Survey design

Detailed survey design is presented elsewhere [27, 28]. Briefly, ENHANCE 2020 was designed to document changes in neonatal mortality rate (NMR) between 2016 and 2020 in Bihar. We estimated a sample of 30,000 live births for ENHANCE 2020, assuming a 10% refusal rate and 85% power to detect a reduction of 18% in NMR from 2016 to 2020 at the 95% confidence level. A multi-stage sampling design was used to obtain a representative sample of births from July 2020 and June 2021 among usual resident women aged 15–49 years births from all 38 districts of Bihar [27]. A total of 267 blocks (50% of the total 534 blocks) were randomly sampled for the survey, which included 187 (70%) blocks with only a rural population and 80 (30%) blocks with both rural and urban populations to reflect the urban-rural population distribution in the state. Within these 267 blocks, the secondary sampling units (SSUs) were villages in rural areas and urban frame survey blocks in urban areas as defined by the Census 2011 [29]. Using systematic random sampling, a total of 1,340 SSUs (941 rural and 399 urban) were sampled in proportion to the number of SSUs in each block.

### Data collection

All households in sampled clusters were enumerated by trained interviewers to document birth outcomes between July 2020 and June 2021 among usual resident women aged 15–49 years. We also documented births between July 2020 and June 2021 for women who had died during or after giving birth to ensure a robust estimation of total births in this population. Relevant to this paper, all women who reported a stillbirth or neonatal death for births between July 2020 and June 2021 were considered eligible for a detailed interview. Women were interviewed in a semi-private place in the household as the trained interviewers requested the household members to provide privacy. Trained interviewers documented socio-demographic information and asked about their post-birth experience. This was done by asking them to respond specifically to questions about – the opportunity to talk about their baby with anyone, availability of support or help in coping with the loss, whether she was blamed for the adverse outcome, whether anyone passed insensitive or hurtful comments, if she was verbally or physically abused, if people avoided to talk to her or meet with her, and if she was excluded from social functions because she was considered inauspicious or unlucky. For all questions where the response was yes, we enquired about the family member(s) involved. These women were also asked if they were specifically asked to forget about the baby, were told that mourning of a baby is taboo and not culturally acceptable, and if were pressured to get pregnant soon. For these questions also, if the response was yes, we enquired about the family member(s) involved. Additionally, the women with a stillborn baby were asked if they wanted to see, hold, and name the baby.

Data were collected between August 2021 and April 2022. The questionnaire was developed in English and then translated into Hindi (local language), after which these were back-translated into English to ensure the accuracy and relevancy of the meanings and intent of the questions. Pilot testing of the questionnaire was carried out, and modifications were made as necessary. Interviews were captured using the Computer-assisted Personal Interview software on hand-held tablets. A total of 20% of interviews were checked in 50% of the 1,340 sampled clusters.

### Analysis

We present the prevalence of a variety of supportive and negative experiences as reported by women with stillbirth and neonatal death by select background characteristics of women. The distribution of women is presented under four categories - those who reported only supportive experience, those who reported only negative experience, those who reported at least one of both supportive and negative experiences, and those who reported neither supportive nor negative experience post their babies’ death. We also present the distribution of people who were reported to provide supportive and negative experiences by women with stillbirth and neonatal death. Distribution of being asked to forget about the baby, not mourn the baby, and being pressured to get pregnant soon are also reported. The distribution of being able to see, hold and name the stillborn are reported for women with stillbirth. The wealth index quartile for each woman, wherein quartile 1 represents the poorest and quartile 4 the richest, in our study were calculated using the standard methods outlined in the NFHS 4 & 5 as detailed by the Demographic Health Survey program for India [30, 31]. We report a 95% confidence interval (CI) for prevalence as relevant. All analyses were performed using STATA 13.1 software (Stata Corp., USA).

## Results

A total of 30,412 births between July 2020 and June 2021 were identified from 261,124 households (91.5% participation), including 582 stillbirths (86.1% participation) and 831 newborn deaths (86.3% participation). The distribution of women who reported stillbirth and neonatal death by select background characteristics is shown in Additional Table 1.

**Fig. 1:**
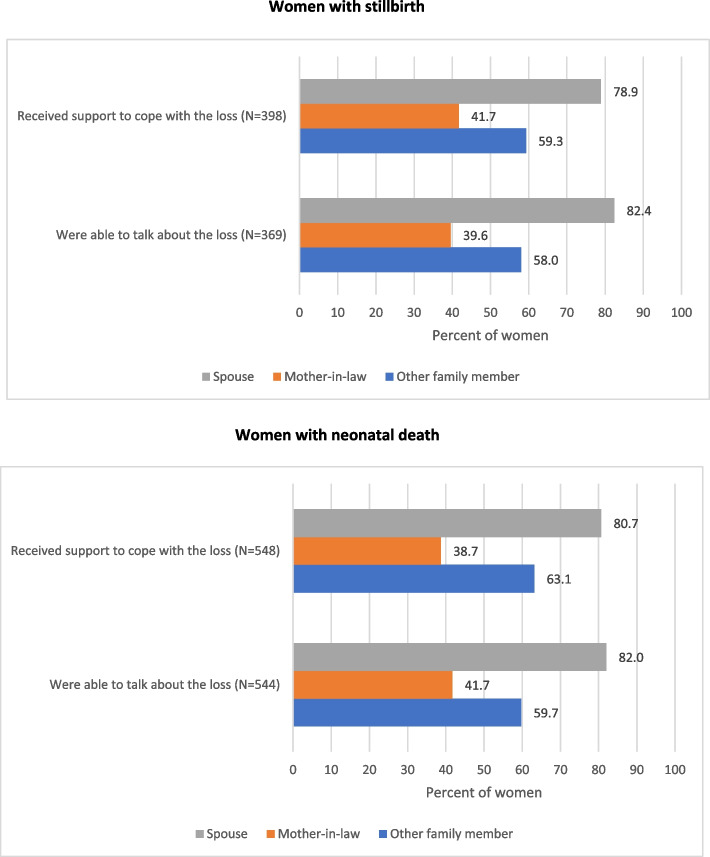
Distribution of family members who provided supportive experience as reported by women with stillbirth and neonatal death. Not mutually exclusive

 Table 1 documents the prevalence of a variety of experiences as reported by women with stillbirth and neonatal death. Data on experience was available for 498 (99.4%) women with stillbirth. The prevalence of supportive experiences of being able to talk to someone about their baby (74.2%; 95% CI: 70.2–77.9 for stillbirths and 76.7%; 95% CI: 73.4–79.7 for neonatal deaths) and for having received support to cope with the loss (80.0%; 95% CI: 76.5–83.5 for stillbirths and 77.3%; 95% CI: 74.0-80.2 for neonatal deaths) was similar for women with stillbirth and neonatal death (Table 1). Both the women with stillbirth and with neonatal death reported their spouse to be the primary person for supportive experience followed by another family member, whereas the mother-in-law was reported only by 38–42% of women (Fig. 1).

**Table 1:** Prevalence of a variety of experiences (not mutually exclusive) as reported by women with stillbirth and neonatal death between July 2020- June 2021 in the state of Bihar

Experience	Number of women with stillbirth* N = 498 (% of *N*; 95% CI)	Number of women with neonatal death* N = 713 (% of *N*; 95% CI)
Able to talk to someone	369 74.2 (70.2–77.9)	544 76.7 (73.4–79.7)
Was extended support/help to cope with the loss	398 80.2 (76.5–83.5)	548 77.3 (74.0-80.2)
*People avoided to talk to her*	55 11.0 (8.6–14.2)	92 12.8 (10.6–15.6)
*People avoided to meet with her*	12 2.4 (1.3–4.2)	22 3.1 (2.0-4.6)
*Received insensitive/hurtful comments about the baby*	93 18.6 (15.4–22.3)	146 20.4 (17.7–23.6)
*Was blamed for stillbirth/death of the baby*	71 14.3 (11.4–17.6)	10 15.0 (12.5–17.8)
*Received verbal abuse for stillbirth/death the baby*	38 7.6 (5.6–10.3)	76 10.7 (8.6–13.1)
*Was physically abused for stillbirth/death of the baby*	8 1.6 (0.8–3.2)	8 1.1 (0.6–2.2)
*Was excluded from activities as she was considered impure/ inauspicious/ unlucky*	8 1.6 (0.8–3.2)	25 3.5 (2.4–5.1)

Statement in italics considered are as negative experience. CI denotes confidence interval

*Data not available for 3 and 4 women with stillbirth and neonatal death, respectively

At least one negative experience was reported by 155 (30.9%; 95% CI: 27.0-35.1) women with stillbirth and 233 (32.5%; 95% CI: 29.1–36.0) with neonatal death. The most commonly reported negative experience was receiving insensitive/hurtful comments about the baby (18.6%; 95% CI: 15.4–22.3 for stillbirth and 20.4%; 95% CI: 17.7–23.6 for neonatal deaths) followed by being blamed for baby’s death (14.3%; 95% CI: 11.4–17.6 for stillbirths and 15.0%; 95% CI: 12.5–17.8 for neonatal deaths) as shown in Table 1. Being avoided to talk was reported by 11.0% (95% CI: 8.6–14.2) for stillbirths and 12.8% (95% CI: 10.6–15.6) with neonatal death. No significant variation was observed in the prevalence of reporting negative experiences by the sex of baby, either for stillbirths or neonatal deaths.

**Fig. 2:**
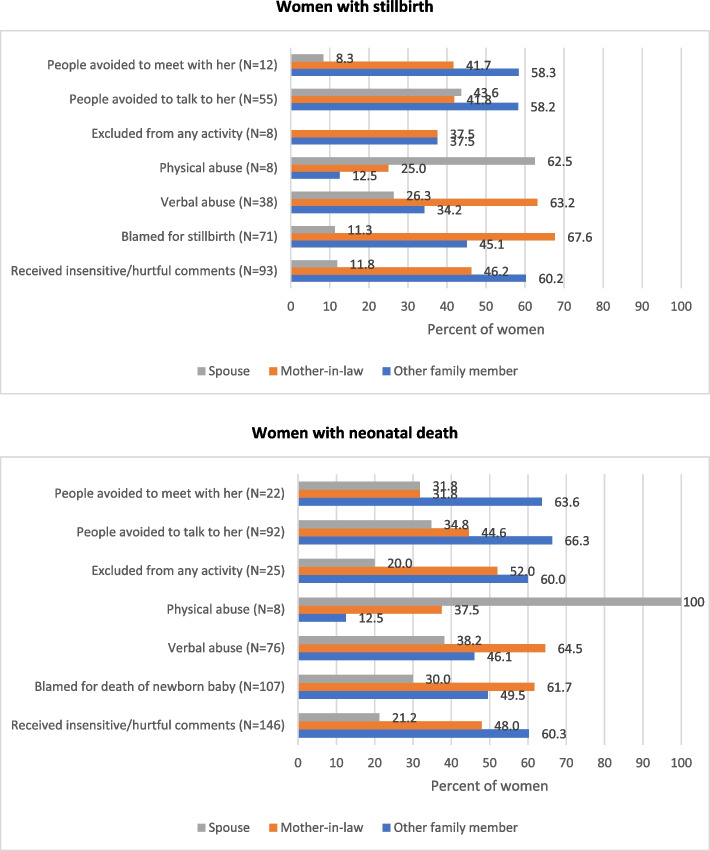
Distribution of family members responsible for negative experience as reported by women with stillbirth and neonatal death. Not mutually exclusive

 Table 2 documents the prevalence of any negative experience reported by women by the select socio-demographic characteristics. The reporting of prevalence of any negative experience was significantly higher for women in the age group of 15–19 years, followed by those aged 20–24 years, for women with neonatal death as compared with the older women. However, this age-related difference was not significant for women with stillbirth. Additionally, women with stillbirth and neonatal death those who were living in urban areas reported higher prevalence of negative experiences compared to women in rural areas, although these differences are not statistically significant. No significant variation was documented for any negative experience based on maternal education and wealth index quartile for women with stillbirth and neonatal death. Overall, other family members were primarily responsible for the experience of avoidance in meeting or talking and insensitive comments, the spouse for physical violence, and the mother-in-law for most of the other negative experiences irrespective of stillbirth or neonatal death (Fig. 2).

Considering women with stillbirth, 320 (64.4%; 95% CI: 60.2–68.6) reported only supportive experience, 146 (30%; 95% CI: 25.6–33.6) reported both at least one supportive and at least one negative experience, 9 (1.8%; 95% CI: 0.9–3.4) reported only negative experience, and 22 (4.4%; 95% CI: 2.7–6.4) reported neither. This distribution for women with neonatal death was 452 (63.7%; 95% CI: 60.1–67.2), 214 (30.2%; 95% CI: 26.9–33.7), 19 (2.7%; 95% CI: 1.7–4.2) and 24 (3.4%; 95% CI: 2.3-5.0), respectively.

A total of 362 (72.7%) and 550 (77.1%) women with stillbirth and neonatal death were asked to forget about the baby as a means of coping mechanism, 9 (1.8%) and 23 (3.2%) were told not to mourn about the loss of the baby, while 21 (4.2%) and 41 (5.7%) were pressurised to get pregnant soon, respectively.

**Table 2:** Prevalence of negative experience reported by women with stillbirth and neonatal death between July 2020- June 2021 in the state of Bihar. CI denotes confidence interval

Background characteristics	Women with stillbirth*		Women with neonatal death*	
	Total *N* = 498	Prevalence of negative experience *N* (% of N; 95% CI)	*N* = 713	Prevalence of negative experience *N* (% of N; 95% CI)
**Overall**	498	155 (30.9; 27.0-35.1)	713	233 (32.5; 29.1–36.0)
**Maternal age**				
15–19 years	42	13 (31.0; 18.8–46.5)	63	25 (39.7; 28.3–52.3)
20–24 years	211	73 (34.6; 28.5–41.3)	338	121 (35.8; 30.8–41.1)
25–29 years	142	38 (26.8; 20.1–34.7)	177	59 (33.1; 26.6–40.4)
30–34 years	70	17 (24.3; 15.6–35.8)	94	22 (23.4; 15.9–33.1)
>=35 years	36	14 (38.9; 24.4–55.7)	44	6 (13.6; 6.2–27.4)
**Maternal education**				
No education	196	59 (30.1; 24.1–36.9)	264	76 (28.7; 23.5–34.4)
Class 1–5	109	25 (30.1; 21.2–40.9)	177	54 (38.3; 30.6–46.6)
Class 6–12	166	66 (34.4; 28.0–41.4)	242	91 (32.7; 27.5–38.5)
More than class 12	29	5 (17.2; 7.2–35.8)	33	12 (36.4; 21.7–54.0)
**Wealth index quartile**^#^				
1	136	38 (27.9; 21.0–36.1)	222	76 (34.2; 28.3–40.7)
2	134	43 (32.1; 24.7–40.5)	184	63 (34.1; 27.6–41.2)
3	112	34 (30.4; 22.5–39.5)	181	52 (28.7; 22.6–35.8)
4	118	40 (33.9; 25.9–42.9)	127	42 (33.1; 25.4–41.7)
**Place of residence**				
Urban	92	32 (34.8; 25.7–45.1)	98	40 (40.8; 31.5–50.9)
Rural	409	123 (30.1; 25.8–34.7)	618	193 (31.2; 27.6–34.9)

*Data not available for 3 and 4 women with stillbirth and neonatal death, respectively

^#^2 cases missing for woman with neonatal death

**Fig. 3:**
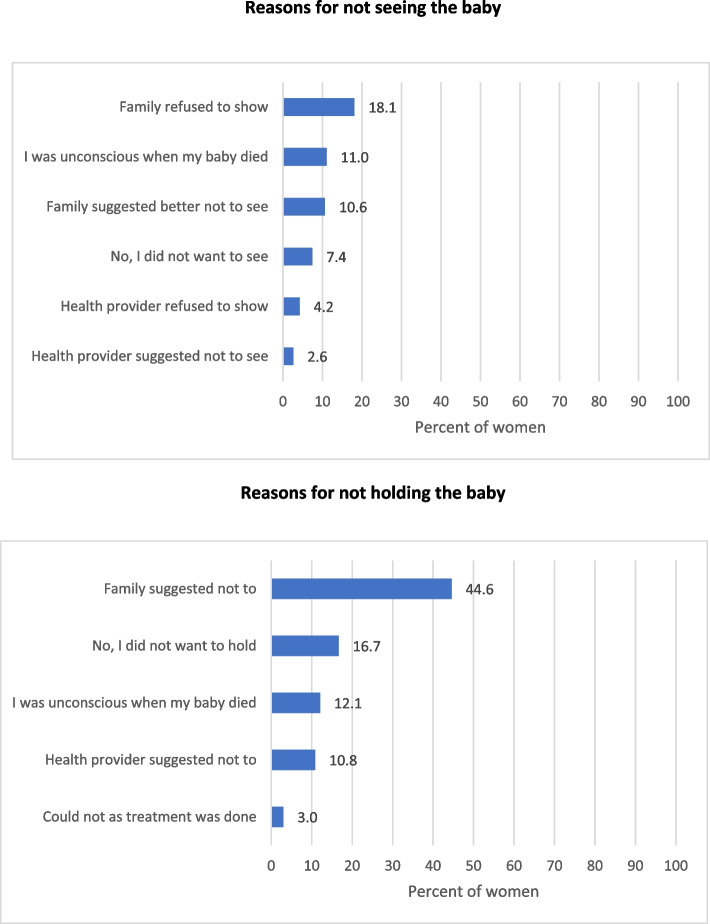
Distribution of reasons reported by women with stillbirth as to why they did not see or hold their baby. Data not available for 3 women

 Among the 498 women with stillbirth, 56 (11.2%) had named their baby, 208 (41.8%) wanted to name their baby but did not, and 150 (30.1%) did not name the baby as there was no practice of naming a dead baby. A total of 229 (45.9%) women with stillbirth reported seeing their stillborn baby, 90 (18.1%) reported that their family had refused to show, and 53 (10.6%) reported that their family suggested it better not to see the dead baby (Fig. 3). Only 64 (12.8%) women with stillbirth reported holding their baby, 222 (44.6%) reported that their family suggested not to hold the baby, and 83 (16.7%) reported that they did not want to hold their baby (Fig. 3).

## Discussion

Nearly three-fourths of women with adverse birth outcomes reported supportive experiences, and one-third reported negative experiences in this population-based documentation of the socio-cultural context in a representative sample of women with stillborn and newborn deaths. The nuanced understanding of the distribution of these experiences by the socio-demographic profile of women is of significant relevance to guide the formulation of strategies to further support women by addressing the negative aspects. Importantly, the extent and type of supportive and negative experiences reported were similar for women with stillbirth and women with newborn death.

Documenting the prevalence of supportive experiences for women with stillbirth or neonatal death is crucial for a complete understanding of the psychosocial aspects of their experiences [8, 26, 32]. With the majority of these women reporting supportive experiences and their spouse as the primary person for this support, it underscores the need for male involvement in the support programs [33, 34]. Male involvement in maternal care has been recognized as the key strategy in improving pregnancy outcomes, [35–41] and our study has highlighted its role in post-partum care including when a baby did not survive. On the other hand, it is important to note that the physical abuse reported by women was perpetrated majorly by their spouses. The prevalence of physical violence during pregnancy predominately by spouse is reported by one in three women in India [42, 43]. A significantly higher proportion of stillbirth has been reported by women with a history of physical violence during pregnancy [44], and women who experience spousal abuse during pregnancy have been reported to have different postpartum contraceptive use patterns as compared to women who were not abused during pregnancy [45]. More work is needed to explore how spouse involvement can be increased throughout the continuum of care for women and ways to decrease the prevalence of physical abuse by spouses are urgently needed in the Indian context.

Women reported less support from mothers-in-law and more negative experiences with them, both for having stillbirth and newborn death. This finding is critical in formulating programs and strategies to improve support for women as mothers-in-law play an essential role in Indian culture in shaping the relationships between the husband and wife [46–49]. The significant role of mother-in-law in intra-household decisions, planning of family size, and antenatal care has been reported from India [50, 51]; however, physical and psychological abuse by mother-in-law is also well documented [52–54]. Relevant research to identify options for more support from the mother-in-law by women post adverse birth outcome is needed, along with identification of the needs of the grieving mothers-in-law post-death of her grandchild [55].

The majority of the women reported that they were asked to forget about their baby in order to cope with their loss, and a small proportion of women reported being pressured to conceive soon in this population. These findings are similar to studies from other countries that have documented disenfranchised grief when parents felt their grief was not legitimised or accepted by health professionals, family, or society [56–58]. Furthermore, bereaved parents have reported persistent psychological distress, being afraid to prepare for birth, and differing emotions in subsequent pregnancies, including volatile emotional states by women [8, 59–62]. As part of the development of a toolkit to promote advocacy for respectful bereavement care following stillbirth among the healthcare providers in India [63], discussions with the bereaved parents highlighted how they felt abandoned by their healthcare provider on diagnosis or delivery of stillbirth, their unmet need for more compassion from their clinician and support staff, and their inability to comprehend how they lost the baby when pregnancy was uneventful thus far. Importantly, this work also documented the limitations as informed by the healthcare providers to deliver compassionate care including lack of experience, lack of resources, excess workload, and limited conversations about stillbirth in general [63]. Health-care providers have a crucial role to play in not only providing support for the grieving parents during this time but also generating a supportive environment by the family through communicating with them the possible physical and psychological implications of loss of a baby on the bereaved parents, in particular the mother [9]. Support from health care professionals is considered important by women in high-income countries [8], and their role and perceptions of providing and enable a supportive environment in developing country settings needs attention [64].

Rituals such as naming, honouring and memorializing help in creating a living bond, which acts as a coping mechanism for the loss of a baby, and spending time with their stillborn by the bereaved parents is also considered a ritual in some countries [8, 65, 66]. Seeing and holding the baby is known to be an effective intervention in high-income settings to improve the wellbeing of both the bereaved parents and family [8, 67–70]. Despite the feeling of grief and loss, mourning in some countries, including in India, is actively discouraged and suppressed by the family and also by health care providers [8, 21]. Findings from our study have reinforced that seeing, holding or naming a stillborn baby is not culturally acceptable for most. This also underscores the need for more locally relevant research to explore if the high-income setting intervention to see and hold the baby can be acceptable in an Indian setting, and also to understand what other culturally appropriate means can be adopted with regards to the baby to improve wellbeing of both the bereaved parents and family [71, 72].

In general, morbidity associated with bereavement is an important public health issue, yet economic and resource investments to effectively implement and sustain integrated bereavement services are sorely lacking at national and global levels [73]. The type of negative experiences reported by women with stillbirth and neonatal death have several important implications for health care practices, societal attitudes, and support systems in the country. India needs to invest in health system to support and respect the affected family to help them recover from their loss. Perinatal mortality counselling program to provide crisis intervention and support for families could be introduced as a pilot intervention in India [74]. With the critical role of spouse and mother-in-law as documented in our study in the type of experience women have post adverse birth outcome have, piloting of the interventions will need to include spouse and mother-in-law to ensure that interventions are grounded in reality for the bereaved women.

## Conclusion

With India accounting for the highest numbers of stillbirths and neonatal deaths globally, one-third of women reporting negative experiences translate into a significant number of women. These population-based data can facilitate in identifying interventions to improve the post-partum experience of women with adverse birth outcomes in India.

## Supplementary Information

Supplementary Material 1.

## Data Availability

All data and materials relevant to the study are included in the article or uploaded as supplementary information.

## Abbreviations

ENHANCE 2020: Every Newborn Health Assessment & Neonatal Care Evaluation 2020
NMR: Neonatal Mortality Rate
CI: Confidence Interval
