# Supplementary material for: Socio-cultural practices and experience of mothers’ post stillbirth and newborn death: a population-based perspective from India
Source: BMC Pregnancy Childbirth. 2024 Nov 25;24:778. doi: 10.1186/s12884-024-06906-0 (PMC11587595; doi:10.1186/s12884-024-06906-0)
Supplement: Supplementary file 1 — Supplementary Material 1. [file 12884_2024_6906_MOESM1_ESM.docx]

Additional Table 1. Distribution of women who reported stillbirth and neonatal death between July 2020 - June 2021 in the state of Bihar by background characteristics.

| **Background characteristics** | **All women irrespective of the birth outcome**  **N=7,270** | **Number of women with stillbirth**  **N=501 (%)** | **Number of women with neonatal death**  **N=717 (%)** |
| --- | --- | --- | --- |
| **Maternal age**^§#^ |  |  |  |
| 15-19 years | 488 | 42 (8.4) | 63 (8.8) |
| 20-24 years | 3197 | 211 (42.1) | 338 (47.1) |
| 25-29 years | 2258 | 142 (28.3) | 178 (24.8) |
| 30-34 years | 905 | 70 (14.0) | 94 (13.1) |
| 35 years & above | 422 | 36 (7.2) | 44 (6.1) |
| **Maternal education***^†¥#^ |  |  |  |
| No education | 2352 | 196 (39.2) | 265 (37.0) |
| Class 1-5 | 1641 | 83 (16.6) | 141 (19.7) |
| Class 6-12 | 2845 | 192 (38.4) | 242 (38.8) |
| More than class 12 | 430 | 29 (5.8) | 33 (4.6) |
| **Wealth index quartile***^†¶#^ |  |  |  |
| 1 | 1892 | 136 (27.2) | 222 (31.1) |
| 2 | 1865 | 134 (26.8) | 185 (25.9) |
| 3 | 1807 | 112 (22.4) | 181 (25.3) |
| 4 | 1700 | 118 (23.6) | 127 (17.8) |
| **Place of residence**^€#*^ |  |  |  |
| Urban | 1307 | 92 (18.4) | 98 (13.7) |
| Rural | 5963 | 409 (81.67) | 619 (86.3) |

^§^ Chi-square test for significance: p=0.152 for stillbirth.

^¥^ Chi-square test for significance: p=0.007 for stillbirth.
^¶^ Chi-square test for significance: p=0.599 for stillbirth.

^€^ Chi-square test for significance: p=0.816 for stillbirth.

^#^ Chi-square test for significance: p<0.001 for neonatal death.
*Chi-square test for significance: p=0.002 for neonatal death.
